# Case-based learning enhances clinical competencies in Thai veterinary students: a mixed-methods approach

**DOI:** 10.4142/jvs.25149

**Published:** 2026-06-26

**Authors:** Adisorn Yawongsa, Suchart Watanachai, Waraporn Aumarm

**Affiliations:** 1 Department of Large Animal and Wildlife Clinical Science, Faculty of Veterinary Medicine, Kasetsart University, Nakorn Pathom 73140, Thailand.; 2 Division of Surgery and Theriogenology, Faculty of Veterinary Medicine, Khon Kaen University, Khon Kaen 40002, Thailand.; 3 Department of Companion Animal Clinical Sciences, Faculty of Veterinary Medicine, Kasetsart University, Bangkok 10900, Thailand.

**Keywords:** Case-based learning, veterinary education, clinical competency, mixed-methods research, pedagogical innovation

## Abstract

**Importance:**

Case-based learning (CBL) represents a promising shift toward competency-based veterinary education, but the evidence from Southeast Asian programs is limited.

**Objective:**

This study evaluated veterinary students' perceptions of structured CBL effectiveness in Thai veterinary education and whether these perceptions varied with academic performance.

**Methods:**

A convergent parallel mixed-methods design was used at the Faculty of Veterinary Medicine, Kasetsart University. Quantitative data were collected from 318 students (88.3% response rate) using a structured 22-item questionnaire across four domains (professional knowledge, communication skills, personal benefits, facilitator performance) on 5-point Likert scales. Groups were compared by Grade Point Average (GPA; 2.01–2.99 vs. > 2.99) using the Mann-Whitney *U* test. Nine focus groups (n = 63) were analyzed thematically.

**Results:**

Students perceived all four domains positively. Mean scores were comparable for professional knowledge (4.08 ± 0.61), facilitator performance (4.06 ± 0.72), and personal benefits (4.03 ± 0.65), and lowest for communication skills (3.89 ± 0.75). Students with a GPA of 2.01–2.99 reported greater perceived benefit than higher-achieving peers in professional knowledge (*U* = 10,109; *p* = 0.036; *r* = 0.14) and personal benefits (*U* = 10,167; *p* = 0.043; *r* = 0.14); differences in communication (*U* = 10,734; *p* = 0.192; *r* = 0.09) and facilitator performance (*U* = 10,783; *p* = 0.213; *r* = 0.08) were not significant. Thematic analysis produced two themes: perceived CBL effectiveness and areas for development.

**Conclusions and Relevance:**

Students perceived CBL as offering meaningful educational value, with lower-performing students perceiving particularly strong benefits, and perceived that CBL supported their clinical reasoning, collaborative learning, and communication. Effective implementation was perceived to require systematic faculty development, case-curriculum alignment, and structured group management. These findings support broader CBL integration while highlighting critical implementation considerations for educational equity.

## INTRODUCTION

Contemporary veterinary education faces mounting pressure to evolve beyond traditional didactic approaches toward competency-based curricula that prepare graduates for complex clinical decision-making [1,2]. The transformation from knowledge-centered to competency-based education reflects the recognition by the profession that effective veterinary practice requires integration of clinical reasoning, communication skills, and collaborative problem-solving abilities [3].

Case-based learning (CBL) is a pedagogical innovation addressing these educational imperatives. Derived from problem-based learning principles, CBL utilizes authentic clinical scenarios to stimulate inquiry-based learning within collaborative small-group environments [4,5]. Unlike traditional lecture-based instruction, CBL positions students as active participants in diagnostic reasoning and treatment planning, fostering the development of clinical competencies essential for professional practice [6]. The theoretical foundation of CBL rests on constructivist learning principles, emphasizing knowledge construction through social interaction and authentic problem-solving experiences [7]. In veterinary contexts, CBL provides students with simulated patient encounters before clinical rotations, allowing a risk-free exploration of clinical reasoning processes while developing confidence in diagnostic and therapeutic decision-making [8,9].

Within veterinary education, research has shown promising outcomes across diverse international contexts. Patterson [10] reported significant increases in veterinary students' self-confidence across three clinical reasoning domains after CBL intervention in the United States. Similarly, studies have documented improvements in communication skills and collaborative learning behaviors among veterinary students participating in CBL programs [11,12]. European implementations have shown comparable results. Malher et al. [11] reported CBL effectiveness in French veterinary education for dairy herd health management, and Duckwitz et al. [13] reported positive student acceptance of case-based blended learning in German veterinary public health education. Australian research by Thurman et al. [12] highlighted the collaborative learning benefits of CBL in veterinary curricula, emphasizing peer-to-peer knowledge exchange.

Nevertheless, CBL implementation is not without challenges. Student readiness for self-directed learning, cognitive load management, and facilitator preparation represent critical factors influencing the educational outcomes [14,15]. Students with limited prior knowledge may struggle to engage with complex clinical cases, while those preferring traditional learning approaches may resist collaborative problem-solving formats [16]. Despite the growing interest in CBL within veterinary education, limited research has examined student perceptions of its effectiveness using rigorous mixed-methods approaches. Understanding student perspectives is crucial for optimizing CBL implementation and addressing potential barriers to learning effectiveness.

The primary research question guiding this investigation was how veterinary students perceive the effectiveness of CBL in developing clinical competencies. Accordingly, a mixed-methods design that captured both quantitative measurements and rich qualitative insights was used to address this question. The specific objectives were as follows: 1) assessing student perceptions across four key domains of CBL effectiveness, 2) identifying the factors influencing perception variations among student cohorts, and 3) exploring the qualitative themes related to CBL strengths and areas for improvement.

## METHODS

### Study design and setting

This mixed-methods study used a convergent parallel design, collecting quantitative and qualitative data simultaneously to provide a comprehensive understanding of student perceptions [17]. The study was conducted at the Faculty of Veterinary Medicine, Kasetsart University, where CBL has been integrated into the curriculum since 2014.

The CBL implementation, facilitation protocols, and assessment methods remained consistent throughout the study period (2014–2019), ensuring comparability of student experiences and perceptions across all cohorts.

### CBL curriculum implementation

The CBL program targets 4th and 5th-year veterinary students, with each cohort encountering 12 carefully selected cases annually across four veterinary disciplines: companion animal medicine (3 cases), large animal medicine (3 cases), farm animal production (3 cases), and veterinary public health (3 cases). The cases were distributed across two academic semesters, with each case requiring two weeks of intensive small-group work.

### Case examples and characteristics

#### Case 1

The cases were designed to reflect authentic clinical scenarios with graduated complexity. In companion animal cases, students assumed the role of veterinarians and received key initial client complaints as the starting point. For example, a companion animal case presented *“Bella,” a 7-year-old spayed female Golden Retriever, with the owner stating: “Bella has not been herself for the past three days - she has been very tired and does not want to eat much.”* Students worked collaboratively to ask targeted questions about the clinical signs and develop initial differential diagnoses based on history-taking. The facilitator, role-playing as the client, provided only the information the students specifically requested, simulating realistic clinical interactions. After gathering a comprehensive history, the students refined their differential diagnoses and then requested specific physical examination findings. They might ask: *“What was her temperature and heart rate?”* or *“What color was the mucus membrane?”* Again, the facilitator provided physical examination findings progressively as the students asked relevant questions, encouraging them to think systematically about body systems.

Based on the history and physical examination findings, the students developed a prioritized list of differential diagnoses, ruling in diseases that aligned with their collected data and ruling out those that did not fit the clinical picture. They then collaboratively decided which diagnostic techniques to use, justifying their choices. The facilitator provided them with the results, such as radiographic images or the raw laboratory data. The students interpreted the result themselves. The case progressed through multiple decision points as the students received diagnostic results and refined their diagnoses, ultimately reaching either a definitive diagnosis or a well-reasoned tentative diagnosis with appropriate treatment plans. This interactive format required students to engage actively in clinical reasoning, collaborate effectively, and communicate their thought processes clearly.

#### Case 2

A farm animal production case presented “Tilapia Farm F3,” a commercial fish farm experiencing acute mortality. The students received the initial complaint: *“We have a tilapia farm with 5 earthen ponds, and we have been losing fish for the past 7 days. It started with 30–50 fish dying per day, but now we are losing over 1,000 fish daily.”*

The students worked collaboratively to gather additional history by asking targeted questions: *“Can you describe the clinical signs you have observed in the live fish?”* The facilitator, role-playing as the farm owner, only provided the information the students requested: *“The fish are swimming slowly, floating at the surface, trying to gulp air, their skin looks pale, and their fins appear eroded.”* The students then requested farm management details, including stocking density (150,000 fish weighing 0.5 g each per pound), water source, feeding practices, and environmental factors. Based on history and clinical observations, the students developed initial differential diagnoses and systematically requested specific diagnostic information. They requested the water quality parameters, received data showing elevated total ammonia nitrogen (6 ppm), and asked permission to examine fish specimens. The students analyzed wet mount preparations showing parasites, necropsy findings revealing pale gills and internal organ changes, bacterial culture results, and microscopic examination of tissue imprints. The case progressed through multiple decision points as the students interpreted diagnostic results, refined their diagnoses, and developed comprehensive management recommendations addressing immediate treatment, water quality management, stocking density adjustments, and prevention strategies. This multifaceted approach required integrating aquaculture management, parasitology, bacteriology, water chemistry, and economic considerations typical of modern aquaculture practice.

All cases were developed by the course coordinator and validated by the course committee to ensure clinical authenticity and the appropriate complexity for student learning levels.

### Facilitator training

Faculty facilitators underwent 6 h of structured training before each academic course, covering CBL pedagogy, guided inquiry techniques, and management of group dynamics. The facilitator pool comprised 12 faculty members from four veterinary departments: companion animal clinical medicine, large animal and wildlife medicine, farm animal production, and veterinary public health. Each case included detailed facilitator guidelines with standardized learning objectives, key discussion points, and suggested questioning strategies. These guidelines enable faculty to facilitate cases outside their primary expertise area, allowing specialists from one discipline to guide cases from other veterinary specialties.

The students were organized into groups of 8–10 members, maintaining consistent group composition throughout the academic year. Each group was assigned a faculty facilitator who guided case discussions for approximately 6 h per case (3 h weekly for 2 weeks). The first week focused on case analysis, problem identification, and diagnostic planning, while the second week emphasized treatment planning and case resolution.

### Participants

The study population comprised all eligible veterinary students (n = 360) enrolled in years 4–6 during the 2014–2019 period. A convenience sampling approach yielded 318 participants (response rate: 88.3%). For qualitative data collection, purposive sampling was used to select focus group participants, ensuring representation across academic years and achievement levels. Nine focus groups were conducted (3 per academic year), each comprising 6–8 students stratified by Grade Point Average (GPA) and gender.

### Data collection instruments

A structured questionnaire was developed based on established CBL evaluation frameworks. The instrument comprised demographic information and 22 items distributed across 4 domains professional knowledge (7 items), communication skills (5 items), personal benefits (6 items), and facilitator performance (4 items). All effectiveness items utilized 5-point Likert scales (1 = strongly disagree to 5 = strongly agree).

Semi-structured focus group guides were developed to explore student experiences in depth, covering the perceived learning outcomes, comparisons with traditional teaching methods, and recommendations for program improvement.

### Data collection procedures

Quantitative data were collected through anonymous online surveys distributed via Google Forms after each academic semester. Focus groups were conducted by trained research assistants independent of the teaching faculty to minimize response bias.

### Statistical analysis

Statistical analyses were performed using SPSS version 28.0 (IBM, USA). Nonparametric analyses were conducted because the data distribution was nonnormal, as confirmed by Shapiro–Wilk tests. Between-group comparisons used the Kruskal–Wallis test for multiple groups, and the Mann–Whitney *U* test for pairwise comparisons. The *p* values < 0.05 were considered significant.

Focus group recordings were transcribed verbatim and analyzed using applied thematic analysis [18]. Three researchers independently conducted open coding, followed by collaborative axial coding to identify the preliminary themes.

## RESULTS

### Participant characteristics

A total of 318 students participated in the quantitative survey: 116 4th-year (36.5%), 103 5th-year (32.4%), and 99 6th-year students (31.1%). The gender distribution was relatively balanced (male: 47.2%, female: 52.8%). The academic performance varied, with 117 students (36.8%) having GPAs of 2.01–2.99 and 201 students (63.2%) achieving GPAs > 2.99.

### Quantitative results

#### Overall CBL effectiveness perceptions

The students reported positive perceptions across all four evaluated domains. The mean scores were professional knowledge (4.08 ± 0.61), communication skills (3.89 ± 0.75), personal benefits (4.03 ± 0.65), and facilitator performance (4.06 ± 0.72), as listed in Table 1.

**Table 1:** Mean rank of student's perception and analysis of the relationship between responding students' perceptions and students' year

Student's year	Number	Professional knowledge	Communication	Personal benefit	Facilitator
4th	116	148.78	160.01	153.80	171.01
5th	103	169.98	171.05	172.41	171.31
6th	99	161.16	146.68	152.74	133.73
χ^2^		2.986	3.535	3.049	11.521
df		2	2	2	2
*p* value		0.225	0.171	0.218	0.003^*^

*Significant at *p* < 0.05.

#### Academic year differences

Significant differences in facilitator performance perceptions were observed across academic years (χ^2^ = 11.521, df = 2, *p* = 0.003). Sixth-year students reported lower satisfaction with the facilitator's performance than 4th and 5th-year students.

#### Academic performance differences

Students with lower GPAs (2.01–2.99) reported significantly higher perceptions of CBL effectiveness in professional knowledge (Mann-Whitney *U* = 10,109, *p* = 0.036, *r* = 0.14) and personal benefits domains (Mann-Whitney *U* = 10,167, *p* = 0.043, *r* = 0.14) compared to higher-achieving peers. The details are listed in Table 2. These findings represent the students' self-reported perceptions of learning benefits and do not indicate objective differences in academic performance or in clinical skill acquisition between GPA groups.

**Table 2:** Analysis of the relationship between responding students' perceptions and student’s average grade

Students GPA	2.01–2.99 (n = 117)	>2.99 (n = 201)	Mann-Whitney *U*	*p* value	Effect size (*r*)
Professional knowledge	173.60	151.29	10,109	0.036^*^	0.14
Communication	168.26	154.40	10,734	0.192	0.09
Personal benefit	173.10	151.58	10,167	0.043^*^	0.14
Facilitator	167.84	154.65	10,783	0.213	0.08

GPA, Grade Point Average.

*Significant at *p* < 0.05.

### Qualitative results

Thematic analysis revealed two primary themes: 1) CBL effectiveness and 2) Areas for development.

### Theme 1: CBL effectiveness

#### Enhanced professional knowledge

The students consistently reported that CBL improved their clinical reasoning abilities and knowledge integration. A fifth-year student explained: *“CBL helped me organize my thinking in a step-by-step manner. Instead of learning subjects separately, we learned to apply multiple subjects together, which is essential for clinical practice.”*

The students particularly valued the multi-species approach of CBL, which provided broader professional perspectives, particularly in farm animal medicine and veterinary public health.

#### Communication and teamwork development

The students reported significant improvements in communication skills through small-group interactions and simulated client communications. The collaborative nature of CBL fostered teamwork skills and peer learning.

#### Clinical preparation

The students recognized the value of CBL in preparing them for clinical rotations. Many 6th-year students reported feeling more confident during actual patient encounters because of their CBL experience.

### Theme 2: areas for development

#### Facilitator consistency

The students identified facilitator variability as a significant challenge. Different facilitators used varying approaches to case discussion, creating confusion about the optimal clinical reasoning processes.

#### Learning environment optimization

Long-term group assignments produced mixed outcomes. Although some groups developed strong collaborative relationships, others experienced conflicts that hindered learning effectiveness.

The primary research question guiding this investigation was how veterinary students perceive the effectiveness of CBL in developing clinical competencies, and whether these perceptions vary based on academic performance levels.

#### Case sequencing and prior knowledge

Fourth-year students particularly struggled with cases requiring knowledge they had not yet acquired, especially in farm animal medicine and veterinary public health.

## DISCUSSION

The positive student perceptions of the impact of CBL on professional knowledge align with established educational theory supporting active learning approaches in health professions education [19]. The students' reports of improved clinical reasoning and knowledge integration reflect the ability of CBL to promote deep learning through authentic problem-solving experiences [20]. These findings mirror international research showing similar outcomes across diverse veterinary and health professions education contexts [21,22].

The finding that lower-GPA students perceived greater benefits from professional knowledge development suggests a particular value of CBL for students requiring additional support. This aligns with research suggesting that scaffolded learning approaches benefit students with varying levels of preparation [23]. The structured group environment of CBL may provide peer support and guided practice that particularly benefits students struggling with independent knowledge integration—a pattern observed consistently across diverse veterinary and health professions education settings [12,21,24]. These findings reflect student perceptions of learning benefits rather than objective measures of skill acquisition or academic performance improvement.

The positive impact on communication skills reported by students reflects the inherent emphasis of CBL on collaborative learning and peer interaction [24]. The small-group format provides repeated opportunities for students to articulate their thinking, defend their reasoning, and engage with alternative perspectives, skills essential for effective veterinary practice. These findings are consistent with research in veterinary and allied health professions education showing similar improvements in communication skills through active, case-based learning approaches [8,12,25].

Nevertheless, the mixed experiences with team development underscore the importance of careful group management and clear expectations for collaborative engagement—a challenge noted across diverse institutional contexts in veterinary education [12,24,26,27]. The tendency for some groups to divide tasks rather than engage in collaborative problem-solving suggests the need for explicit teamwork training and facilitator guidance in promoting effective group dynamics, regardless of the educational setting.

The identification of facilitator variability as a primary concern reflects a common challenge in CBL implementation across health professions education globally [28]. Effective CBL facilitation requires specific skills in guided inquiry, management of group dynamics, and formative assessment—competencies that may not align with traditional lecture-based teaching approaches [29]. This challenge has been documented consistently across veterinary and health professions education programs internationally, suggesting a universal need for comprehensive facilitator development [22,27,28].

Research in medical education across multiple countries has shown that structured facilitator training programs can significantly improve the effectiveness of CBL [30]. International best practices from leading veterinary schools underscore the importance of ongoing faculty development that focuses on consistent learning objectives, guided inquiry techniques, and strategies for managing diverse student learning needs within group settings [22,27,28].

These findings contribute to growing international evidence supporting the effectiveness of CBL in veterinary education. Studies in veterinary and health professions education have documented similar benefits in clinical reasoning and communication skills through CBL [12,21], while European programs reported comparable outcomes in specialized areas such as farm animal medicine, veterinary public health, and preclinical curriculum integration [11,13,22]. The consistency of findings across diverse cultural and educational contexts suggests that the fundamental pedagogical principles of CBL transcend national boundaries.

Several practical implications emerge from the best international practices and the study findings:

1) Comprehensive facilitator development: Programs worldwide should invest in extensive facilitator training focusing on CBL pedagogy, guided inquiry techniques, and consistent learning objective achievement [22,28,30].
2) Strategic case selection: Case selection should align carefully with the students' prior knowledge while providing appropriate challenge levels for meaningful engagement [13,22,27].
3) Adaptive group management strategies: Clear expectations for collaborative engagement and strategic group rotation may optimize teamwork development and prevent stagnant group dynamics [12,24].
4) Cultural adaptation: Although core CBL principles remain consistent internationally, successful implementation requires adaptation to local educational cultures and student learning preferences [5,8,27].

Several limitations warrant consideration when interpreting these findings. First, data collection from a single institution limits generalizability to other veterinary education contexts with different curricular structures or student populations. Second, reliance on student perceptions rather than objective learning outcome measures limits the conclusions about actual skill development. The cross-sectional design prevents an assessment of longitudinal changes in student perceptions or long-term learning outcomes. In addition, although the present findings align with international research trends, a direct comparison with other educational contexts would strengthen generalizability claims.

Cross-cultural validation studies comparing CBL effectiveness across different educational systems and cultural contexts would enhance the understanding of universal versus culture-specific implementation factors. International collaborative research examining the outcomes of CBL across multiple veterinary schools would provide valuable insights into optimal implementation strategies and could identify universal best practices.

Research incorporating objective measures of clinical reasoning development, such as script concordance tests or standardized patient encounters, would complement perception-based findings and provide more robust evidence of learning outcomes. Investigations of specific facilitator characteristics and training approaches associated with optimal student outcomes could improve facilitator development programs globally.
